# Recruiting and consenting into a peripartum trial in an emergency setting: a qualitative study of the experiences and views of women and healthcare professionals

**DOI:** 10.1186/s13063-016-1323-3

**Published:** 2016-04-11

**Authors:** Julia Lawton, Claire Snowdon, Susan Morrow, Jane E. Norman, Fiona C. Denison, Nina Hallowell

**Affiliations:** Centre for Population Health Sciences, University of Edinburgh, Edinburgh, UK; Department of Medical Statistics, London School of Hygiene and Tropical Medicine, London, UK; MRC Centre for Reproductive Health, University of Edinburgh, Edinburgh, UK; Ethox Centre, Nuffield Department of Population Health, University of Oxford, Oxford, UK

**Keywords:** Peripartum trial, Informed consent, Emergency situation, Recruitment, Ethics

## Abstract

**Background:**

Recruiting and consenting women to peripartum trials can be challenging as the women concerned may be anxious, in pain, and exhausted; there may also be limited time for discussion and decision-making to occur. To address these potential difficulties, we undertook a qualitative evaluation of the internal pilot of a trial (Got-it) involving women who had a retained placenta (RP). We explored the experiences and views of women and staff about the information and consent pathway used within the pilot, in order to provide recommendations for use in future peripartum trials involving recruitment in emergency situations.

**Methods:**

In-depth interviews were undertaken with staff (*n* = 27) and participating women (*n* = 22). Interviews were analysed thematically. The accounts of women and staff were compared to identify differences and similarities in their views about recruitment and consent procedures.

**Results:**

Women and staff regarded recruitment as having been straightforward and facilitated by the use of simplified (verbal and written) summaries of trial information. Both parties, however, conveyed discordant views about whether fully informed consent had been obtained. These differences in perspectives appeared to arise from the different factors and considerations impinging on women and staff at the time of recruitment. While staff placed emphasis on promoting understanding in the emergency situation of RP by imparting information in clear and succinct ways, women highlighted the experiential realities of their pre- and post-birthing situations, and how these had led to quick decisions being made without full engagement with the potential risks of trial participation. To facilitate informed consent, women suggested that trial information should be given during the antenatal period, and, in doing so, articulated a rights-based discourse. Staff, however, voiced opposition to this approach by emphasising a duty of care to all pregnant women, and raising concerns about causing undue distress to the majority of individuals who would not subsequently develop a RP.

**Conclusions:**

By drawing upon the perspectives of women and staff involved in the same trial we have shown that they may operate within different experiential and ethical paradigms. In doing so, we argue for the potential benefits of drawing upon multiple perspectives when developing information and consent pathways used in future (peripartum) trials.

**Trial registration:**

ISCRTN 88609453.

## Background

Multicentre randomised trials frequently encounter difficulties meeting their recruitment targets, resulting in extensions to recruitment periods and delays to reporting and implementing findings [1, 2]. This has led to the development of a substantial body of research that has explored the challenges of recruitment and has demonstrated that recruitment processes can be influenced by a complex mix of factors. For instance, it has been shown that the way in which trial information is presented to potential participants, including the language and terminology used, can shape their understandings of a trial [3], and can impact on ‘opt-in’ levels [4, 5]. Such research has led to the development of tools to promote effective recruitment processes [6]. Understanding of ways in which the clinical setting of a trial can impact on experiences of decision-making has also increased, with studies highlighting the range of contexts and circumstances in which individuals are asked to consider participation, from emergency settings [7–9], where decision-making may be deferred [10, 11], to those where there is plenty of time for reflection and discussion [12].

Research suggests that recruitment and informed consent processes can also be influenced by staff perceptions and experiences. For example, staff can find recruitment difficult when there are too many competing demands on their time [13], and may be reluctant to approach those they perceive as vulnerable [14, 15]. In addition, staff may decide not to approach some eligible people due to their own lack of equipoise with regards to particular groups or individuals [16], or concerns about compromising an on-going therapeutic relationship [17]. These kinds of issues, considerations and concerns often prove to be more complex and challenging than anticipated at a trial’s outset. Thus, as trials move into new clinical areas and address novel questions, new and unforeseen issues are likely to arise.

It has also been shown that information and consent pathways used in recruitment processes may not necessarily lead to a clear and lasting understanding of a trial’s purpose, design and implications [18, 19]. This has raised concerns about whether fully informed consent can be gained, especially in trials involving recruitment of potentially vulnerable individuals or those who are eager to access resources or treatments, which they cannot afford or cannot access through routine clinical care [20–23].

It has been suggested that recruiting and consenting to peripartum trials can create particular challenges and difficulties because such trials involve approaching women who may be anxious and in pain, and could be experiencing mental, emotional and physical exhaustion [24]. Potential participants may be subject to the effects of analgesics which may further compromise their ability to understand trial information and, hence, to give fully informed consent [25–27]. In some intrapartum and peripartum trials there may also only be a very limited time period in which discussion and decision-making can take place due to the time-critical nature of the diagnosis and management of the life-threatening condition being studied [24, 25, 28]. These kinds of problems are similar to those identified in neonatal intensive care trials where recruitment can also take place shortly after birth. In this setting, women and their partners have been shown to vary in their reactions to research participation, with some being very clear about their course of action and others describing their struggles with the information they were given and the choices they were required to make [29].

In recognition of the above and other potential difficulties surrounding recruitment and consenting into perinatal trials, we undertook a qualitative evaluation of the internal pilot of the Got-it trial: a double-blind randomised trial involving women who have a retained placenta. Our key aim was to explore the experiences and views of women and trial staff about the information and consent pathway used within the pilot with close attention paid to the potentially challenging context within which recruitment took place. Our objectives were to provide recommendations that might be used to refine the information and consent processes used in the main Got-it trial; and, to offer insights which could be used to inform the information and consent pathways used in future peripartum trials.

## Methods

### The research setting

The Got-it trial is a randomised, placebo-controlled, double-blind, pragmatic UK-wide randomised controlled trial (RCT) involving women who have a retained placenta (RP) recruited from delivery wards in UK maternity hospitals. RP is a major cause of postpartum haemorrhage and affects around 2 % of vaginal deliveries in the UK. It is diagnosed when the placenta is not delivered within 30 minutes following active management or 60 minutes after physiological management followed by active management of the third stage of labour after delivery of the baby [30]. The current definitive management of RP is manual removal of the placenta, which is a surgical procedure requiring trained personnel and an operating theatre [30]. The aim of the Got-it trial is to determine whether use of glyceryl trinitrate (GTN) spray, as compared to a placebo, can facilitate delivery of the placenta without having to undertake manual or surgical delivery in theatre. GTN is a drug which was originally developed for the prevention and relief of angina attacks. Its side-effects include headache, dizziness, flushing/feeling hot, a drop in blood pressure or a rise in pulse rate. In the clinical context of RP, it could also affect blood loss due to its primary mode of action as a muscle relaxant.

The trial’s inclusion and exclusion criteria are listed in Table 1. During the pilot, once a diagnosis of RP had been made, potential recruits were approached by a delegated and trained member of the clinical or research team. These women were given written information in the form of a one-page summary leaflet accompanied by a detailed participant information sheet. The trial team developed the summary information because it was felt that, due the emotional and/or physical impact of birth, women might find the full information sheet too lengthy and burdensome to read. RP is also a potentially life-threatening complication with the amount of blood loss increasing the longer a placenta is retained. Provision of summary information was, therefore, also considered appropriate to facilitate timely consent and definitive management of the RP if it was not delivered following administration of the trial drug. Women were also given a verbal explanation of the trial that covered all the elements in the participant information sheet and consent form. Women who gave their consent were randomised to receive GTN or a placebo spray, which they self-administered under their tongue (two puffs). The placebo spray was designed to be identical in taste and appearance to GTN so neither participants nor staff could determine the outcome of randomisation. Women whose placentas were not delivered within 15 minutes were taken to theatre for manual removal of the placenta under regional or general anaesthesia, with the method of anaesthetic being determined by the clinical team and being dependent on the urgency of need for placental delivery.

**Table 1:** Trial inclusion and exclusion criteria

Inclusion criteria
Women with a retained placenta
Women aged 16 or over
Women having a vaginal delivery (including women with a previous caesarean section)
Haemodynamic stability (systolic blood pressure more than 100 mmHg and pulse less than 110 beats per min)
Over 14 weeks’ gestation
Exclusion criteria
Unable to give informed consent
Suspected placenta accreta/increta/percreta
Multiple pregnancy
Women undergoing an instrumental vaginal delivery in theatre
Allergy or hypersensitivity to nitrates or any other constituent of the formulation
Taken alcohol in the last 24 hours
Concomitant use of phosphodiesterase inhibitors (such as sildenafil, tadalafil, or vardenafil)
Contra-indication due to one of the following: severe anaemia, constrictive pericarditis, extreme bradycardia, incipient glaucoma, glucose-6-phosphate dehydrogenase deficiency, cerebral haemorrhage and brain trauma, aortic and/or mitral stenosis and angina caused by hypertrophic obstructive cardiomyopathy, circulatory collapse, cardiogenic shock and toxic pulmonary oedema
Currently participating in another CTIMP

The trial, as already indicated, comprises two phases: an internal pilot followed by a substantive RCT. The pilot commenced in October 2014 and involved eight sites that entered the pilot in a staggered way. Its purpose was to provide evidence and reassurance of the feasibility and effectiveness of all trial processes, including those relating to recruitment and obtaining informed consent. In order to progress to the substantive RCT, the pilot had to reach a target of 75 recruits within the first 7 months of recruitment. This target was exceeded with 87 women having been recruited by the end of April 2015; very low decliner rates were also reported across the pilot sites, with only six of those approached choosing not to take part. The substantive trial is now underway and is due to be completed in 2017.

### Qualitative study design

In order to gain multiple perspectives on the information and consent pathway used in the trial’s pilot phase, both trial-recruited women, women who declined participation, and recruiting staff were invited to take part in the qualitative study. Data were collected by means of in-depth interviews because these afforded the flexibility needed for participants (staff and women) to raise and discuss issues which they perceived as salient, including those unforeseen at the study’s outset [31, 32]. The use of one-to-one interviews also afforded privacy, allowing participants to share personal experiences and negative views about the trial’s recruitment and consent procedures, should they chose to do so. The study employed an iterative approach that entailed simultaneous data collection and analysis [33]. This allowed the areas explored in the interviews to be revised in light of emerging findings. Interviews with women and staff were undertaken in parallel, enabling issues raised by one participant group (e.g. women) to be explored in interviews undertaken with the other group (e.g. staff).

### Sample and recruitment

Recruitment was undertaken in all eight centres involved in the pilot study and took place throughout the pilot’s duration (i.e. from November 2014 to April 2015) to accommodate the staggered entry of sites into the pilot and allow for the inclusion of participants from all sites. Women were approached within 2–3 days of having taken part in the trial and were either given a recruitment pack while in hospital, or if they had already been discharged, a pack was posted out to them. Staff were given or sent recruitment packs. In both cases, an ‘opt-in’ procedure was used and recruitment materials made potential participants aware that the team conducting the interviews were independent from the clinical trial team.

Women were purposively sampled so there was diversity in the final sample in terms of age, education/occupation, parity and ethnicity (see Table 2). Staff were selected for interviews if they had been involved in trial delivery, recruitment or consenting of the women. Across the centres, these staff comprised obstetricians, research midwives and midwifery staff. All participants gave their written informed consent to take part in an interview.

**Table 2:** Participant characteristics – women

	Number	%
Age (years)		
Mean: range	30.7	18–40
< 30 years	8	36
Ethnic group		
White British	17	77
South-east Asian	2	9
Other	3	14
Highest education level		
School	7	32
Further education	2	9
Degree	8	36
Higher degree	5	23
Previous birthing experiences		
Primagravidae	9	41
Previous retained placenta	5	39

### Data collection and analysis

Interviews were conducted by NH who has over 20 years of experience of carrying out qualitative interviews on sensitive topics. To reduce potential problems with recall bias, women were interviewed within 4 weeks of having taken part in the pilot trial. While women were given the choice of a phone or face-to-face interview, virtually all opted to be interviewed by phone; most staff also opted for a phone interview. No obvious differences were observed between the content and quality of interviews undertaken face-to-face and those done by phone. Interviews were informed by topic guides developed in the light of literature reviews, inputs from staff and lay advisors, and focussed upon: women’s experiences of birth; the views of women and staff about the trial and trial procedures; the method, timing and content of information delivery; and, the information and consent pathway used during the trial’s pilot phase (see Table 3). As indicated above, the topic guides were also revised in light of emerging findings. For example, in the initial phase of interviewing, women and staff were all asked a very general question about the timing of information-giving in the pilot – *What do you think about the timing of the information given in the Got-it trial*? After most women spontaneously volunteered that they would have appreciated receiving information about the trial during their pregnancy, the staff topic guides were expanded to explore their views about providing such information during the antenatal period. Interviews with women averaged around 25 minutes and those with staff 45 minutes. In all but two cases (where women had to end the interview abruptly to attend to their babies) all key areas in the topic guides were covered and explored in depth. Data collection continued until data saturation was achieved; that is, until no new findings or themes could be identified in new data collected.

**Table 3:** Areas of questioning explored in the topic guides

Topic guide – Women	
• Demographic information	
• Experiences of labour, birth and postpartum period	
• Experience of consent pathway used in the Got-it trial	
• Timing of approach for participation in the Got-it trial	
• Understanding of trial and information	
• Factors influencing decision to participate in Got-it	
• Information needs	
• Experiences of trial participation	
• Views on research participation in general	
Topic guide – Staff	
• Demographic information	
• Former trial experience	
• Perceptions of the Got-it trial (equipoise)	
• Involvement in the Got-it trial	
• Recruitment and consenting experiences in the Got-it trial	
• Perceptions of the consent pathway used in the Got-it trial	
• Timing of approach for participation in the Got-it trial	
• Participants’ understanding of trial and information	
• Quality of participants’ consent	
• Impact of research on HCP-patient relationships	

All interviews were digitally recorded and transcribed in full. As already indicated, data analysis was iterative and commenced as soon as data collection began. Data were analysed thematically by JL and NH using the method of constant comparison [34]. This thematic approach entailed individual interviews being read through repeatedly before being cross-compared to identify issues and experiences cutting across different accounts. A comparative analysis of women’s and staff accounts was also undertaken to identify differences and similarities in their views about recruitment, consent procedures, and the timing of information delivery − and the reasons for these. JL and NH undertook independent analyses before meeting to discuss and reach agreement on key findings and themes and to develop a coding frame. The qualitative analysis software package NVivo9 (QSR International) was used to facilitate data coding and retrieval. Coded datasets were subjected to further, in-depth analysis to identify additional themes and illustrative quotations.

Ethical approval for the trial and the qualitative research was given by Newcastle and North Tyneside 2 Research Ethics Committee. To safeguard confidentiality, all participants were allocated pseudonyms which are used below.

### Implementation group

An implementation group was put together for this study comprising members of the trial team, staff representatives from each of the pilot trial sites, patient representatives, the qualitative study team (JL, NH and CS) and a member of the Trial Steering Committee. The findings reported below were fed back to this group at the end of the pilot phase of the trial. In light of these, the group compiled a series of recommendations to facilitate the running of the substantive trial, some of which focussed upon refining the information/consent pathway. These are returned to later in the paper.

## Results

Forty-nine women and 37 staff were invited to participate. Twenty-two (45 %) women and 27 (73 %) staff were interviewed and full details about the sample can be found in Tables 2 and 4. Although it had been our intention to interview ‘decliners’, four of the six women who declined trial participation during the pilot were deemed by clinical staff to be inappropriate to approach, and the remaining two did not opt-in to the qualitative study.

**Table 4:** Participant characteristics – healthcare professionals

	Number	%
Staff^a^ (*n* = 27)		
Doctors	10	37
Consultants	3	11
Clinical midwives	6	22
Labour ward leads	2	7
Research midwives	11	41
Highest education level		
Professional qualification/diploma	2	7
Degree	17	63
Higher degree	8	30
Years in post		
< 2 years	6	22
2–5 years	16	59
> 5 years	5	19
No previous trial experience	7	26

^a^Doctors were interviewed from seven sites, clinical midwives from four and research midwives from all eight sites. At least two staff members, a research midwife plus one other, were interviewed in seven sites

In keeping with quantitative data from the pilot (which showed good levels of recruitment and very low decliner rates) both women and staff presented their recruitment experiences as having been relatively straightforward and uncontroversial. However, while the trial was considered easy to recruit into, views about the consenting procedures and, more specifically, the timing of the information needed to promote fully informed consent were more mixed, with notable differences between women and staff. Below, we consider these findings in more detail. We begin with women’s perspectives and experiences of the information and consent pathway used in the pilot and how they thought it might be improved, before moving onto staff accounts. In doing so, we attempt to understand how, and why, the differences in their perspectives arose.

### Women’s views about the trial

In general, women conveyed very positive views about the trial despite, in most cases, having subsequently had to go to theatre for manual removal of the placenta. Women described the trial as being a valuable and well-intended piece of research and which, as Helen suggested, will ‘hopefully mean that women in the future won’t need to go to theatre’. They also pointed out that the trial had been very straightforward in terms of what had been required of them: ‘I was happy to do it, ‘cause I thought “it’s only 15 minutes to wait, to see if it (the spray) works”’ (Hazel), and had entailed only very minimally invasive procedures: ‘…like putting a spray under my tongue, that didn’t bother me at all’ (Kirsty). In addition, most women suggested that they had welcomed the opportunity to try an alternative intervention rather than going straight to theatre:

‘I think it made me thankful that they’d offered me an alternative rather than just whisking me off to theatre and sort of knocking me out or anything like that. It made me feel like they were giving me all of the possible options for the scenario that I was in. So I felt like they were looking out for (me), not only for my health, but psychologically as well.’ (Trina)

As we will now go onto consider, as well as the simple nature of the intervention (a spray) raising no disincentive to participate, women’s earlier experiences of labour and delivery were important components underlying their positive views about the trial. We will also show how these earlier experiences informed women’s preferences for information delivery as well as leading to many later questioning whether their consent had been fully informed.

### Women’s views about information delivery and giving informed consent

In the interviews, women were invited to talk about the events that had led up to their taking part in the trial. While this line of questioning prompted a minority (*n* = 3) to describe a relatively straightforward and pain-free birth, many recounted birthing experiences which had been painful and often protracted and, for which, in some cases, analgesics had been required:

‘It was really horrible. The pain was unbearable, it really was totally unbearable for me. I was feeling like “I will die of this thing.”’ (Amal, prescribed diamorphine)

‘I’d already been in labour for about 30 hours, I was in a lot of pain so I’d asked for an epidural… But then several hours later – and my waters had broken at this point – the epidural had come out slightly at the back so I ended up without pain relief for the pushing part, which was really painful.’ (Faith)

As well as having been left physically and emotionally exhausted by these kinds of births, some women, including Tracy below, highlighted additional distress which had resulted from a complicated delivery and ensuing concerns about their baby’s health and safety:

‘I started off on the labour on Sunday, it was like a really, really slow labour, contractions just were never, ever going to deliver a baby. So they induced me on the Tuesday and within like half an hour my contractions were like every 6 and 10, which were just too quick, my body couldn’t handle it, I don’t think I could handle it. So they slowed my labour down. I had diamorphine and whatever else I could have. When I went to the labour ward I had to have the clip on baby’s head to check her heart because obviously she was struggling with the contractions speeding up again. I just thought it was just horrendous. I was so worried about her (baby) and so scared.’ (Tracy)

In some cases, quick births were also described as having had adverse physical and emotional impacts, with Kirsty, for instance, sharing her experiences of having gone into shock after a rapid and intense labour:

‘I’d been very vocal during the birth and then afterward I went really quite quiet. Because everyone was like, “are you sure you’re ok, are you alright?” and they kept on checking I was ok… I think because it happened all in all it was a quick birth, I think I was in a bit of shock about that.’ (Kirsty)

Women also described how their distress had intensified after discovering that they had a RP. Notably, worry and panic reactions were shared by those, such as Trina, who had no prior knowledge of this type of condition and who had assumed that it must be very serious:

‘You know when somebody says that there’s something wrong and you’re not expecting there to be, and you don’t know a lot about it, there were moments that it was quite scary.’ (Trina)

### Women’s experiences of, and views about, the information and consent pathway

Given their pre-birth experiences, most women described how, by the time they were approached to take part in the trial, they had been ‘tearful and emotional and exhausted’ (Anna), ‘overwhelmed’ (Arlene) and ‘at the end of my tether’ (Hannah). As a consequence, women described how they had valued being given a succinct information leaflet to read rather than a full participant information sheet, because, as Faith explained, ‘at that point I was cross-eyed and it was just about all I could take in’ and, as Celia elaborated, ‘it was easy to understand, because I was not, you know, probably not my normal self… I wasn’t up to being bamboozled or overwhelmed with information or anything’. Others described how they had simply been too exhausted and distracted to read even the succinct written materials; and, hence, how it had been very helpful to have had staff present to go through the literature with them and provide verbal descriptions and summaries of the trial.

Thus, women were generally very positive about the content and mode of delivery of trial information which, as Kate described, was ‘as good as it could have been, given everything which was going on at the time’. However, most also noted that, by the time they had been approached to take part, they had simply been too exhausted, distracted and/or emotionally overwhelmed to assimilate and retain all the information provided:

‘I genuinely think they probably told me everything about the trial, but my head was elsewhere, I was utterly exhausted.’ (June)

‘…they (possible side effects of taking GTN) were on the form but if I’m being honest, I cannot remember what any of them were. I remember that the nurse read them out and went through it with me but I was utterly done in.’ (Faith)

Some women, including Heather, also reflected on how their concentration and ability to take trial information on board had been further compromised by their awareness that they were in an emergency situation with a lot of activity taking place around them:

‘I think I had the baby and I think the midwife were kind of fiddling with the cord and trying to get things out and then they were chatting to me… they were putting the, eh cannula in my hand and stuff as well. So I can’t remember all of it… and I was quite anxious already, thinking am I going gonna have to get this epidural done?’ (Heather)

In addition, most described recognising, with hindsight, that the approach to take part in the trial had taken place at a time when they had been in a very vulnerable emotional situation; one which had resulted in their consenting from ‘a point of desperation’ as Arlene aptly put. The women who felt this way described how, at the time they were recruited, they would have considered almost any option which might have prevented them from having to undergo further invasive medical procedures and from having to leave their baby:

‘And she said that I could try this new drug and it’s the last resort before an epidural to take the placenta out. So I just thought, “oh my God I don’t want an epidural” so I tried it.’ (Hannah)

‘I’d have done anything to sort of avoid having to go to theatre. My daughter had been taken into the SCBU* because she was premature, so I wanted to get to her as quickly as possible. And obviously the quicker I delivered the placenta, the quicker would get to my daughter.’ (Trina)

(*SCBU special care baby unit)

As a consequence of being in what they saw as a desperate state, most of these women described having made their decision to take part in the trial more or less instantly, without clarifying or seeking further information or consulting others:

‘I just said yes straightaway. I didn’t want to mess around, I just wanted to hold my baby really… It was just me, snap decision, saying “yeah, right, ok”, I don’t think I even looked at my husband.’ (Liz)

‘At time when I said yes, I didn’t need to think about… all I knew is that I was a mum without a baby who needed me.’ (Trina)

Some women also reflected on how, due to their eagerness for a quick-fix solution which might prevent them from going to theatre, they had neither been interested in, nor receptive to, learning about the possible risks and side effects of taking GTN:

‘But yeah, you know, I didn’t really think at that point, it was just so appealing to me that I could have a spray and, you know, wouldn’t have to go to theatre that I probably didn’t want to think about the risks too much.’ (Susie)

Hence, while nearly all women were confident that they had actively consented to take part in the trial and that their consent had been ‘given freely’ as June put it, the majority also questioned whether, with hindsight, their consent had been fully informed:

‘At the time I felt I was quite informed. I felt fine about taking part. I didn’t feel worried about it. I know I was, it was okay… But looking back, there was quite a lot going on. I don’t know how much I was taking on board, so I am not sure how informed my decision really was but, at the time, I don’t think I cared either.’ (Diane)

In general, women did not consider their participation to have been problematic despite questioning whether their consent had been fully informed. This, as indicated above, was in large part due to their positive perceptions of the trial and the minimal demands that participation had required. However, there was a minority who conveyed more ambivalent views, all of whom who went on to experience a postpartum haemorrhage or another complication which they thought might have been due to taking GTN:

‘I just had a massive haemorrhage then sort of straight away… within 2 minutes of having it (trial spray), my BP dropped and my heart rate shot up… and there was all just suddenly doctors in the room… and I’m thinking, “oh my God, what have I done, I’m bleeding to death.”’ (Lynne)

‘I mean I had a really bad panic attack, it was really intense, my heart rate was through the roof for quite a while… And was so ill afterward, you feel like “oh God, was that the right choice that I made”, you know it scared me a bit that I took the choice without really thinking about it.’ (Hannah)

As the quotes from Lynne and Hannah also make apparent, these women conveyed regret and concern about having made a decision to take part at speed, and without fully considering the risks.

### Women’s views about improving the information and consent pathway: extending information-giving into the antenatal period

As women’s accounts made apparent, the difficulty with giving and gaining informed consent arose from the context and timing of the recruitment approach and, relatedly, their physical, emotional and mental states at the time. For this reason, when asked what they thought about the trial’s information and consent pathway, most spontaneously suggested that their decision-making and, hence, ability to give informed consent could potentially have been enhanced had they been exposed to trial information before they went into labour. Specifically, women described how earlier exposure to this information might have allowed them to digest and reflect upon it at a time when they were better placed to assimilate the details:

‘I found that, because I’d just given birth, I wasn’t in the right mind to understand what people were saying to me, so I think it would have been better to have got the information before.’ (Kate)

Some also suggested that earlier provisioning of information might have enabled them to consult others as well as to draw upon thinking and preliminary decision-making made at a time when they were not in a desperate and vulnerable state. As Susie, like others, speculated, this might have helped to prevent making a rapid decision in an anxious and panicked state:

‘I think, I mean… “earlier” as in before the labour, kind of during my pregnancy with midwives… because then if I would have been approached with it, you know, me and my husband could have maybe talked about it beforehand and said, you know “if I did need the spray, would I have it? You know, rather than make a quick decision based on panic.”’ (Susie)

A minority of women, however, did question the merits of providing trial information to all expectant mothers given the relative rarity of RPs:

‘When you’re preparing to give birth there’s such a small chance that you’ll, you know, have a retained placenta, I think if there’d been more information available I may have looked at it but I don’t think I really would have given it much thought beforehand.’ (Celia)

While Celia questioned the efficacy, for her, of receiving information during the antenatal period, she was keen to emphasise that information should be made available to all expectant mothers so they could decide for themselves whether or not to engage with it:

‘I am a firm believer that you should have all the information available if you want to, you know, look into things – I definitely think it should be down to the mother’s choice.’ (Celia)

In keeping with Celia’s comments, most women indicated that they would have welcomed this choice because, for them, the antenatal period had been a time when they had been ‘hungry for information’, as Alice put, in order to be able to make informed decisions. Indeed, several women described how they had actively sought out this kind of information because they considered discussion of birthing complications to be a necessary and important part of birth planning:

‘Like I say I’d done a lot of homework into, you know, into the third stage and everything like that, so I did know that could go wrong, I was aware of it, and I was aware that there are ways of dealing with it before you have to go to theatre.’ (Susie)

‘I’m all for that the more information – knowledge is power as far as I’m concerned. So the more that I know the more that I feel comfortable… So I read every – every possible scenario (both laugh) that could have possibly happened in the birthing room.’ (Liz)

### Women’s views about improving the information and consent pathway: extending information-giving into the postnatal period

While most of the women recommended improving the information and consent pathway by introducing information about the trial during the antenatal period, some also highlighted the benefits of revisiting this information during the early postnatal period. This was particularly important for women who did not recall being given or who had lost the full participant information sheet. These women described how they had welcomed receiving copies of trial documentation prior to their discharge from hospital as this had allowed them to revisit and digest information they had not been able to retain at the time of recruitment:

‘I think it was quite handy that I could take that home to read through at some point. Because giving me anything to sort of read, or consent to when I was in the delivery ward was – was pretty pointless, to be honest, because I had no idea what was going on.’ (Trina)

For some women, the desire for more information in the postnatal period extended beyond the provision of formal trial information. Specifically, the minority of women who experienced a PPH or other difficult symptoms (see above) highlighted the benefits of having a debriefing session with trial and/or clinical staff, and conveyed gratitude when they had had the chance to do this. These women described debriefing sessions as presenting important opportunities to revisit their experiences of trial participation with staff and to learn more about the intervention drug, in order to better understand if the problems they had experienced could be explained by having had a RP or if they might have been due to possible exposure to GTN. As these women also indicated, they had wanted this information as they were seeking reassurance that, in the event of a future birth, they would be unlikely to have a similar, traumatic experience:

‘Maybe a little bit of after care in terms of someone coming back and talking me through it, does that make sense? Because obviously I’d been to theatre, I’d had the panic attack it hadn’t worked. And after I did, like I said, I did have a few questions, like I thought, “could it have been the spray that caused my blood pressure to go high?”’ (Shari)

‘Especially after having had such a bad experience (postpartum haemorrhage), it was nice while sort of an inpatient to have some feedback and just sit and talk through it afterwards, you know, with the person who approached me in the first place… Because it worries me, if I did have another one, is it going to happen again?’ (Lynne)

### Staff views and experiences of recruitment and gaining informed consent

Mirroring the accounts given by women, staff described the trial as having been easy to recruit into, due to the possible benefits to women of taking part (avoiding theatre) and the minimal time and effort that participation required:

‘I think it kind of sells itself, I mean I can understand why we don’t get many declining because I would find it hard to find somebody who would say “no, I would rather I went to theatre and have this horrible procedure where someone puts their hand right up inside me and pulls my placenta out.” So why would someone go through that as opposed to sort of, you know, doing that?’ (MW J)

While the trial was seen as an ‘easy sell’ (Dr B), staff also reflected upon the difficulties and challenges of having to recruit and consent women within a time-pressured situation. Staff also noted the additional challenges arising from having to consent individuals who might be tired, distressed and in pain and, hence, have limited ability to concentrate:

‘It’s an interesting environment to consent patients for a clinical trial. Because it’s quite different to a sort of, you know, sit down clinic, have a think about something, then write to me if you’re interested. That’s the thing, it’s a kind of now or never scenario.’ (Dr E)

‘I think it is difficult because by definition somebody who has just had a vaginal delivery is, therefore, exhausted and also now has a complication. Now it may not be immediately life-threatening, but obviously they’re aware there is a problem, so they’re anxious about that.’ (Dr G)

Hence, staff, including those quoted above, reflected on how they had felt an ethical mandate and responsibility to convey information about the trial in clear, succinct and accessible ways, in order to achieve understanding amongst the women they were recruiting. To do this, staff discussed how they had tended to simplify and give ‘the minimum of information’ (Dr J) they thought was needed to gain informed consent:

‘They just need to know the essentials, like the basics, like it’s a trial; it’s a research trial… there’s a spray we can give you, two sprays under your tongue and one is the GTN spray and one is a fake, and we don’t know which is which. I think just the basics, because at the time of having a baby, there’s a lot of things going round in a woman’s head when they’ve had a baby and you don’t remember a lot of what’s going on.’ (MW N)

Staff also highlighted the benefits of delivering information verbally and of using the summary rather than the full version of the trial information sheet:

‘Because the women are under the influence of drugs, tiredness, exhaustion, emotional, you know, it’s important to read the information sheet to them… Because the women, they don’t, in my opinion, have the ability to read the information themselves and retain everything.’ (MW H)

‘There is a leaflet that’s really detailed and I think, you know, if they’ve had opiates and stuff was well they won’t be in the mood for reading that, will they. There’s a simplified version which is much more straightforward and I think this is probably they level you should be aiming for when someone’s just had a baby. I don’t mean that in a condescending way, I just mean they are exhausted and they’ve often been up for 2 days so giving them too much information is unfair really.’ (DR F)

In general, staff saw their attempts as having been successful, despite the challenging circumstances in which recruitment had been undertaken. This was due in large part to what they saw as their own effort and ability to convey information clearly and succinctly, wherein: ‘I’ve managed to do it but with difficulty’ (Dr I). In addition, staff described having determined that informed consent had been given by the women they had recruited based on their observations that these individuals had generally been able to present an understanding and rationale for taking part:

‘…because they usually seem to sort of spell out their rationalisation for why they went for it, you know, “anything that might give even the smallest chance of me going to theatre.”’ (Dr F)

‘You know from what they’ve said, the kind of general consensus was “for the sake of it being so quick, you know, so quick, it’s worth a shot.”’ (MW J)

### Staff views about the timing of information delivery and extending the consent pathway

Given their perception that they had been successful in recruiting and consenting women, most staff conveyed general satisfaction with the information and consent pathway used. Indeed, while staff did acknowledge the challenges of giving women information about the trial in the period immediately following diagnosis of a RP, they all saw this as the most appropriate time to do so:

‘Although broaching an entirely new subject about a trial when they’ve just has a baby is not the optimum time to give them information… I guess it’s as good a time as any in that you know, sometimes I guess the women are just so overwhelmed with all the information they get in pregnancy, to speak to all women about this trial is just a bit too much.’ (Dr B)

‘I don’t think there is an alternative, because the alternative would be to talk to everyone who is pregnant about this may happen and it’s really not that relevant to the majority of the population.’ (Dr H)

Indeed, staff were reluctant to consider other approaches, a position which did not change when individuals were told, during their interviews, that women had expressed a wish for earlier information-giving. To justify their position, most staff framed their opposition in terms of an ethic and duty of care to all pregnant women, wherein the potential (if any) benefits of delivering information during the antenatal period would be outweighed by what they saw as significant emotional costs to recipients:

‘Sometimes ignorance is bliss, you know, women, they just have so much information given to them now throughout their pregnancy and I’m a bit kind of like “is it right to scare them about something that might never happen? Because they’re already terrified about labour.”’ (MW J)

Some of the concerns staff expressed about antenatal information-giving also related to logistical and cost considerations, wherein:

‘If you stop to tell everybody about this trial, but only a couple of percent will have it (a retained placenta), it’s gonna waste so many resources.’ (Dr J)

Other concerns related to efficacy, with some staff suggesting that, even if information was made available to women during the antenatal period, most individuals would fail to engage with it, again making this an inappropriate use of staff effort and resources:

‘I just don’t think it would necessarily be much help… they’re going to take in what they think is relevant and at that time they’re not planning on having a retained placenta, so, I just don’t think it will register with them.’ (Dr E)

Staff did, however, highlight the potential benefits of revisiting trial information and offering a post-trial debriefing which, as MW N suggested, would allow women to better understand ‘what was happening because their head’s a bit more together’. Others, including Dr J, noted that, in their institution, a broadly similar practice was already made available to women who underwent emergency procedures such as a ventouse delivery or a caesarean section wherein: ‘We see our patients a day or two after the procedure to explain to them what has happened and to see if they’ve got more questions’.

## Discussion

There is a growing body of work looking at consent issues in perinatal, neonatal and other trials requiring recruitment to be undertaken in emergency situations, and this study contributes to this literature by reporting the perspectives of patients (women) and staff involved in the same trial. By exploring the recruitment and consent encounter from these dual perspectives, we have revealed a complex picture in which both points of convergence and divergence emerge. Both women and staff agreed that giving and processing information about a trial at the time of a diagnosis of a RP is difficult. Furthermore, both parties saw use of simplified trial materials (e.g. written and verbal summaries) as an important means of imparting information to women when their ability to concentrate might be compromised and there is limited time in which to undertake recruitment. However, while staff maintained the view that the recruitment/consent strategy used in the Got-it pilot was efficacious and acceptable, women, whilst noting that their consent had been given freely at the time, raised retrospective concerns about whether they had actually been able to make a fully informed decision. To understand the differences in these perspectives, the different factors impinging on women and staff at the time of recruitment, and what they considered the key ethical considerations to be, need to be considered.

When undertaking recruitment at the time of an obstetric emergency, staff placed emphasis on information-giving and promoting understanding. In the main, they saw informed consent as having been achieved, in large part because of their perceived ability to impart information about the trial in clear, simple and accessible ways, and their assessments that women had understood the information they had been given. While women also valued clear and simple information at the time of recruitment, they drew attention to the experiential realities of their pre- and post-birth situations and how these also influenced their decision-making. Like the women in the pre-term labour trial studied by Kenyon et al. [24], they described the context in which their recruitment had been undertaken as having been inherently pressurised. In particular, women discussed how their eagerness to avoid going to theatre and their desire to remain with their babies had resulted in rapid decision-making and what they recognised, in hindsight, to be a lack of engagement with the potential risks involved in trial participation. Similar findings have been reported by Snowdon et al. [35], who found that parents involved in perinatal trials also tended to make rapid decisions; in their case, due to fear, panic and concerns about their baby’s safety.

To address their (retrospective) concerns about whether fully informed consent had been given, women highlighted the potential benefits of receiving information prior to giving birth, a finding also reported by Ayers et al. in a study involving parents who agreed for their babies to be recruited into a neonatal trial [36]. These benefits included having the opportunity to learn about the trial at a time when they might be better placed mentally and emotionally to consider the information provided. Women also suggested that receipt of information during the antenatal period could enable them to make preliminary decisions when they were not in a vulnerable situation and to seek, and engage with, the views of others, such as their partners. The adaptations to the information and consent pathway that women proposed, however, were not in accordance with what staff considered feasible and appropriate. In this study, and by virtue of bringing the perspectives of staff and women together, we identified two potential sources of difference. These related to the nature of the information to be given, and perceptions of the impact of that information.

Staff saw antenatal information about the management of RP as difficult and potentially distressing and felt that women should be spared these details unless they became relevant to their situation. While staff generally accepted the value of discussing RPs and the trial after the event, they viewed this information as irrelevant to the majority of pregnant women, due to the relative rarity of RPs. This view, in turn, led staff to suggest that the effort involved in delivering information during the antenatal period would be a misguided and ineffective use of staff time and resources. These views contrasted with those of women who described information gathering during pregnancy (including information about obstetric complications) as a key part of their self-education and preparation for birth. Indeed, in suggesting that information should be made available during pregnancy, most of these women, like those interviewed by Snowdon et al. [37] who had experienced a life-threatening postpartum haemorrhage, presented themselves as ‘information hungry’ and as wanting to be empowered. While some agreed that they might not have found information about RP personally salient, they did not think it was inappropriate for pregnant women to be given this information. Indeed, many women thought it was important for individuals to be able to decide for themselves whether or not to engage with it.

The women in this study thus not only appeared to be drawing upon different experiential paradigms to those invoked by recruiting staff, they also seemed to appeal and make recourse to different ethical discourses. While women presented a rights-based ethical justification, staff drew upon an ethical position in which they emphasised their duty of care to all pregnant women. Specifically, staff weighed the potential benefits to the minority of women who would go on to meet trial inclusion criteria against the potential costs to all pregnant women, who, they suggested, might find information about RPs and the trial distressing and burdensome. In doing so, staff also drew upon a resource-based (ethical) discourse in which they voiced concerns about whether the delivery of trial information during pregnancy would be a prudent use of (scarce) staff time and resources.

### Implications for intrapartum and peripartum research

The accounts of the women who took part in this study offer powerful empirical endorsement for the information and consent pathway developed by Vernon et al. [25], and subsequently enshrined within the Royal College of Obstetrician and Gynaecologists’ (RCOG) guidelines [38], for use in intrapartum trials. Due to a lack of formal clinical guidelines at the time, Vernon et al. developed a pathway for subsequent use in a trial which also involved recruitment of women with a RP. To develop this pathway, they conducted a consultation exercise with consumer groups (local and national consumer groups, local experts in the field of consent issues and members of an ethics committee). This consultation led to the decision by Vernon et al. not only to deliver trial information at the time of recruitment, but also to present information during the antenatal period (in the form of an information sheet given at a booking appointments accompanied by tabloid style brochures distributed in antenatal clinics and labour wards). This method of information delivery was also backed up with posters, publicity in the local press and links to a study website, the intention being to raise awareness and to give women the option of obtaining more information should they wish to do so [25]. Such a strategy fits well with the needs articulated by the women who took part in our study, who were able to draw upon their actual experiences of having been recruited into an intrapartum trial. Indeed, the women we interviewed described both a need and right to access trial information during pregnancy, in order to be able to decide for themselves whether or not to engage with it.

By also drawing upon staff perspectives, this study highlights potential challenges to implementing an information pathway in the antenatal period − challenges which may also apply to other trials recruiting women with rare obstetric complications, such as postpartum haemorrhage [28]. Staff resistance to delivering information during pregnancy could be explained in part by their perceptions that women had given properly informed consent at the time of recruitment. Such a finding mirrors Ferguson’s (2003) observation that staff involved in recruiting into trials of all types are generally confident that they have given appropriate levels of information to enable patients to make informed decisions [39]. However, as described above, our findings also suggest that staff resistance to imparting information during the antenatal period could be due to their operating within different ethical and experiential paradigms to the women. This observation has important implications for the design of information and consent pathways in future trials, especially if this involves consultation with one kind of user group (e.g. patients) but these pathways then require implementation by another group (e.g. health professionals). In light of our own findings, we would recommend that careful thought be given to the constitution of consultation groups, to ensure the views of all parties involved in the recruitment and consent process are taken into account. What is interesting to note in this study is that when the findings outlined above were presented to the implementation group (comprising both health professionals and patient representatives) at the end of the pilot phase, a compromise position was reached. This involved targeting antenatal information at women who are identified as being at increased risk of a RP (e.g. due to having had a previous RP [40]). In addition, the implementation group advised cascading general information about the trial through posters in antenatal clinics and community bases, and via NHS websites and social media feeds, thereby allowing ‘information hungry’ women the opportunity to learn about the trial and access more detailed information should they wish do so. See Table 5 for further details of these key recommendations.

**Table 5:** Excerpts from *Recommendations of the Implementation Group*, May 2015

It is ‘…recommended that the pathway to be used in the main trial should draw upon the principles of the pathway developed by Vernon et al. but be “scaled down”. It was proposed that information about the trial should be displayed in settings where women receive their antenatal care in the form of posters and leaflets. It was also agreed that these documents should contain clear information about how women could access further information should they wish to do so; for instance, by providing contact details of trial staff and links to websites (Got-it or local Trust websites) containing more information about the trial and about retained placentas). The potential to use of social media such as Twitter and Facebook was also highlighted. It was suggested that the proposed method of information delivery would meet the needs of women who are “information hungry” whilst not overburdening those who are not.’
It is ‘…recommended that those women who are identified as being at increased risk of having a retained placenta (e.g. due to having had one previously) should, when possible, be targeted during the antenatal period and these individuals should be given a trial information sheet.’

While the pathway recommended by Vernon et al. [25] and subsequently by the RCOG [38], places heavy emphasis on antenatal information delivery, the women and staff who took part in our study also highlighted the benefits of information-giving being extended into the early postpartum period. In most cases, this was to allow women to revisit information about the trial and learn more about the details at a time when they were better placed physically and emotionally to assimilate and comprehend it. However, there was also a minority of women who had experienced a postpartum hemorrhage or another distressing complication and who expressed a need to discuss their experiences and revisit trial information with staff in order to make better sense of what had happened to them. This need for a debrief has also been expressed by women who have experienced a postpartum haemorrhage or other severe birthing complications in non-trial situations [36, 41, 42] and who described feeling abandoned and left with questions if this was not offered to them [42]. What is particularly salient to note here is that, even if women feel satisfied with consent procedures at the time of recruitment, their perspectives may subsequently change. Not only does this highlight the challenges for staff about making assessments about women’s competency to make an informed decision at the time of recruitment, it also highlights the importance of following up women post trial, especially those identified as having had negative experiences.

A key area of agreement between women and staff lay with the benefits of using summary and verbal versions of trial information at the time of recruitment. The use of this kind of approach is not currently highlighted in RCOG guidelines for obtaining a valid consent for participation in research while in labour or in the immediate postpartum period [38]. Arguably, however, it should be considered for use in future trials involving recruitment of participants experiencing physical and/or emotional exhaustion, especially those where there is only limited time to undertake recruitment. Not only is this a relatively ‘low cost’ option to implement, the staff who took part in our study described how that they had found summary information easy and appropriate to deliver.

### Strengths and limitations

A key strength of this study is that it drew upon the perspectives and experiences of women (trial participants) as well as those of recruiting staff and, in doing so, it has been possible to identify discrepant and sometimes contradictory views. In addition, and in contrast to the study by Vernon et al. [25], these perspectives and experiences were informed by involvement in a real rather than a hypothetical trial scenario. As such, this study offers new and potentially important insights relevant for future trial designs. However, while women recommended that trial information should be delivered during the antenatal period, the current study did not present an opportunity to evaluate whether, and how, pregnant women who are not yet sensitised to the issues around RPs might engage with, and use, this information in practice. This is an important area for future research, as others have also recommended [37]. While this study also provides powerful endorsement for information to be given at the time of recruitment in simplified verbal and written forms, the relatively straightforward (and hence easy to explain) nature of the Got-it trial intervention needs to be taken into account. Hence, future research could evaluate use of simplified/summary information in more complex intrapartum trials as well as in other trials involving recruitment in emergency situations.

## Conclusion

This qualitative study of patients’ and staff experiences of recruitment to a peripartum trial suggests that while gaining and giving consent to research participation in an ‘emergency’ situation may be perceived as relatively straightforward, the consent obtained may not be as informed as it could be. Women’s suggestion that the quality of informed consent could be improved by receiving trial information during the antenatal period was not generally supported by staff on the grounds that this would be burdensome for both individual women and healthcare systems. As the findings of this study suggest, these discrepant perspectives and views may be due to the two parties (women and staff) drawing upon, and making recourse to, different ethical and experiential paradigms. In doing so, we have highlighted the potential importance of including multiple perspectives when developing information and consent pathways for use in future (peripartum) trials.

### Consent

Written informed consent was obtained from participants for publication of their individual details and accompanying quotes in this manuscript. The consent form is held by the authors and is available for review by the editor-in-chief.

## Abbreviations

GTN: glyceryl trinitrate
RCT: randomised controlled trial
RP: retained placenta
RCOG: Royal College of Obstetricians and Gynaecologists
SCBU: Special Care Baby Unit
UK: United Kingdom
